# *Helicobacter pylori* HP0018 Has a Potential Role in the Maintenance of the Cell Envelope

**DOI:** 10.3390/cells13171438

**Published:** 2024-08-27

**Authors:** Kyle Rosinke, Vincent J. Starai, Timothy R. Hoover

**Affiliations:** 1 Department of Microbiology, University of Georgia, Athens, GA 30602, USA; krosinke@uga.edu (K.R.); vjstarai@uga.edu (V.J.S.); 2 Department of Infectious Diseases, University of Georgia, Athens, GA 30602, USA

**Keywords:** *Helicobacter pylori*, lipoprotein, outer membrane vesicles

## Abstract

*Helicobacter pylori* is a bacterial pathogen that colonizes the human stomach, where it can cause a variety of diseases. *H. pylori* uses a cluster of sheathed flagella for motility, which is required for host colonization in animal models. The flagellar sheath is continuous with the outer membrane and is found in most *Helicobacter* species identified to date. HP0018 is a predicted lipoprotein of unknown function that is conserved in *Helicobacter* species that have flagellar sheaths but is absent in *Helicobacter* species that have sheath-less flagella. Deletion of *hp0018* in *H. pylori* B128 resulted in the formation of long chains of outer membrane vesicles, which were most evident in an aflagellated variant of the Δ*hp0018* mutant that had a frameshift mutation in *fliP*. Flagellated cells of the Δ*hp0018* mutant possessed what appeared to be a normal flagellar sheath, suggesting that HP0018 is not required for sheath formation. Cells of the Δ*hp0018* mutant were also less helical in shape compared to wild-type cells. A HP0018-superfolder green fluorescent fusion protein expressed in the *H. pylori* Δ*hp0018* mutant formed fluorescent foci at the cell poles and lateral sites. Co-immunoprecipitation assays with HP0018 identified two enzymes involved in the modification of the cell wall peptidoglycan, AmiA and MltD, as potential HP0018 interaction partners. HP0018 may modulate the activity of AmiA or MltD, and in the absence of HP0018, the unregulated activity of these enzymes may alter the peptidoglycan layer in a manner that results in an altered cell shape and hypervesiculation.

## 1. Introduction

*Helicobacter pylori* is a Gram-negative bacterium (i.e., diderm) that belongs to the phylum Campylobacterota. *H. pylori* colonizes the human stomach, where it can cause peptic ulcer disease and chronic gastritis [1,2,3]. In addition, *H. pylori* is a major risk factor for gastric cancer and mucosa-associated lymphoid tissue lymphoma [4]. Several virulence factors have been identified in *H. pylori*, many of which are lipoproteins that have roles in adhesion [5,6], virulence [7,8], and cell migration in gastric cancer cells [9].

As with other diderms, *H. pylori* release outer membrane vesicles (OMVs), which are nanoparticles derived from the outer membrane that range in size from 20 to 300 nm and are part of normal bacterial growth both in vitro and in vivo [10]. The composition of OMVs has been described in several bacterial species and includes phospholipids, lipopolysaccharide, outer membrane proteins, periplasmic proteins, and peptidoglycan, as well as cytoplasmic components, including DNA and RNA [11,12,13,14,15,16,17]. OMVs have reported roles in a broad range of physiological processes, including cell-to-cell communication, nutrient acquisition, quorum sensing, horizontal gene transfer, interbacterial killing, toxin delivery, and the stress response [10].

*H. pylori* uses a cluster of polar flagella for motility, which is required for colonization in model animal systems [18,19]. The *H. pylori* flagellum is encased in a membranous sheath that is contiguous with the outer membrane, a feature that is shared with many other *Helicobacter* species [20]. In a previous study, we identified a number of protein homologs that are prevalent in *Helicobacter* species that have flagellar sheaths (FS^+^ species) but are absent or underrepresented in *Helicobacter* species that lack flagellar sheaths (FS^−^ species) [21]. One of the proteins found in FS^+^ *Helicobacter* species but absent in FS^−^ *Helicobacter* species is HP0018, a predicted lipoprotein of unknown function. *Campylobacter jejuni* Cj0089 is a predicted lipoprotein of unknown function and a homolog of HP0018. The two proteins share 27% amino acid identity over 82% of their lengths, and both proteins contain a tetratricopeptide repeat (TPR) motif. TRP-containing proteins participate in a variety of cellular processes, including cell cycle control, transcriptional regulation, mitosis, protein transport, protein folding, and regulation of steroid receptor function [22,23].

To determine if HP0018 had a role in flagellar sheath formation, we deleted the *hp0018* homolog in *H. pylori* B128 and characterized the phenotype of the resulting mutant. The original Δ*hp0018* mutant was aflagellated due to a single nucleotide deletion in the homopolymeric tract of cytidines in *fliP*, which encodes a component of the flagellar type III secretion system that transports axial components of the flagellum (e.g., rod, hook, and filament proteins) across the cell membrane [24]. The aflagellated Δ*hp0018* mutant produced large amounts OMVs (i.e., hypervesiculated) that tended to form long chains at the cell pole. Flagellated Δ*hp0018* cells in which *fliP* had reverted to the ‘on’ phase appeared to have a normal flagellar sheath, suggesting that HP0018 is not required for sheath formation. In addition to being hypervesiculated, cells of the Δ*hp0018* mutant were less helical in shape compared to wild-type cells. Co-immunoprecipitation assays identified the peptidoglycan hydrolases MltD and AmiA as potential HP0018 interaction partners. We hypothesize that HP0018 regulates the activity of MltD or AmiA, and the unregulated activity of these enzymes in the absence of HP0018 affects the structure of the cell envelope.

## 2. Materials and Methods

### 2.1. Bacterial Strains and Growth Conditions

*E. coli* NEB^®^ Turbo (New England Biolabs, Ipswich, MA, USA) was used for cloning and plasmid construction. *E. coli* strains were grown in lysogeny broth (LB) broth or LB agar medium supplemented with kanamycin (30 μg/mL), ampicillin (100 μg/mL), isopropyl β-D-1-thiogalactopyranoside (IPTG; 0.1 mM), or 5-bromo-4-chloro-3-indolyl β-D-galactopyranoside (40 μg/mL) when appropriate. *H. pylori* strains used in the study were derived from *H. pylori* B128, which was kindly provided by Dr. Richard M. Peek, Jr. For the routine growth of *H. pylori* strains, the cultures were grown in an atmosphere consisting of 10% CO_2_, 6% O_2_ and 84% N_2_ at 37 °C on tryptic soy agar supplemented with 5% heat-inactivated horse serum (TSA-HS). Liquid cultures of *H. pylori* were grown in brain heart infusion (BHI) medium supplemented with 5% heat-inactivated horse serum with shaking in serum bottles in an atmosphere consisting of 5% CO_2_, 10% H_2_, 10% O_2_, and 75% N_2_. Kanamycin (30 μg/mL) and sucrose (5% wt/vol) were added to the medium when appropriate. The motility of *H. pylori* strains in a soft agar medium consisting of Mueller–Hinton broth, 10% heat-inactivated horse serum, 20 mM 2-(N-morpholino) ethanesulfonic acid (MES; pH 6.0), and 0.4% noble agar was assessed as described [25]. Reagents used for growth media were purchased from Thermo Fisher Scientific (Waltham, MA, USA).

### 2.2. Constructing the H. pylori Δhp0018 Mutant 

Genomic DNA (gDNA) from *H. pylori* B128 was purified using the Wizard genomic DNA purification kit (Promega, Madison, WI, USA) and used as the template for PCR applications. PCR primers used in the study are listed in Table S1, as are the plasmids and *H. pylori* strains generated in the study. DNA sequences corresponding to a 533 bp region upstream and a 530 bp region downstream of the *hp0018* homolog were amplified from *H. pylori* B128 gDNA using PrimeSTAR DNA polymerase (Takara Bio, San Jose, CA, USA) together with the primer pairs 51/52 and 53/54, respectively. Primers 52 and 53 are complementary and introduced Xho1 and Nhe1 restriction sites for subsequent cloning of a kan^R^-*sacB* cassette. The resulting amplicons were joined together by overlapping PCR using Phusion DNA polymerase (New England Biolabs, Ipswich, MA, USA). The overlapping PCR product was incubated with Taq DNA polymerase (Promega, Madison, WI, USA) to add A-overhangs to the 3′-ends of the amplicon, which was then ligated into pGEM-T Easy (Promega) to generate plasmid pKR12. Insertion of the overlapping PCR product into the plasmid was confirmed by DNA sequencing (Eton Biosciences, Research Triangle Park, NC, USA). A kan^R^-*sacB* cassette from plasmid pJC038 [25] was introduced into the Xho1 and Nhe1 sites of plasmid pKR12 to generate plasmid pKR14.

The suicide vector pKR14 was introduced into *H. pylori* B128 by natural transformation, as described [25]. Transformants in which *hp0018* had been replaced with the kan^R^-*sacB* cassette by homologous recombination were enriched by selecting for kanamycin resistance. Using the primers 51 and 54, several kanamycin-resistant isolates were checked by PCR to verify that the kan^R^-*sacB* cassette was integrated into the *hp0018* locus. One of the strains in which *hp0018* was replaced with the kan^R^-*sacB* cassette, which was designated strain H13, was transformed with the suicide vector pKR12. Transformants in which the kan^R^-*sacB* cassette was replaced with the unmarked deletion of *hp0018* resulting from homologous recombination between plasmid pKR12 and the chromosome were counter-selected on TSA-HS supplemented with 5% sucrose, as described [26]. Sucrose-resistant isolates were screened for kanamycin-sensitivity on TSA-HS supplemented with kanamycin, and the deletion of *hp0018* in kanamycin-sensitive isolates was confirmed by PCR using primers 51 and 54 and DNA sequencing of the resulting amplicon. A *H. pylori* B128 strain in which *hp0018* was deleted was designated as strain H19 and stored at −70 °C.

### 2.3. Construction of an Expression Vector for a HP0018-sfGFP Fusion Protein

A gene encoding a superfolder green fluorescent protein (sfGFP) described by Dinh and Bernhardt [27] and codon optimized for *H. pylori* was synthesized by Azenta Life Sciences (South Plainfield, NJ, USA). Primers 102 and 103 were used to amplify the coding region of *hp0018* minus the stop codon along with 500 bp of upstream sequence from *H. pylori* B128 gDNA. Primer 102 contained an overhang that included a NheI site, and primer 103 was complimentary to the 5′-end of the sequence encoding the sfGFP. Primers 104 and 105 were used to amplify the sequence encoding the sfGFP. Primer 104 was complimentary to a portion of primer 103, and primer 105 contained an overhang that included a BamHI site. The resulting amplicons were ligated together, and after adding A-overhangs to the 3′-ends, the overlapping PCR product was ligated into pGEM-T Easy to generate plasmid pKR31. Plasmid pKR31 was digested with NheI and BamHI (New England Biolabs, Ipswich, MA, USA), and the DNA fragment bearing the *hp0018*-*gfp* fusion and upstream region was ligated into pHel3 [28] to generate plasmid pKR40.

### 2.4. Construction of c-Myc Tagged HP0018

To facilitate construction of a c-Myc-tagged HP0018 in *H. pylori*, we modified the shuttle vector pHel3 [28] to allow for the introduction of the c-Myc epitope at the C-terminus of the protein. The modified plasmid, which we designated pHel3-Myc, contains the *fliF* promoter upstream of the tandem BspQ1 sites for Golden Gate cloning followed by a sequence encoding a flexible linker, the c-Myc epitope, and the DDDDK epitope. Primers 206 and 207 were used to amplify *hp0018* from *H. pylori* B128 gDNA. The primers introduced BspQ1 sites immediately upstream and downstream of the start and stop codons of *hp0018*, respectively. The resulting amplicon and plasmid pHel3-Myc were digested together with BspQ1 and ligated using Fast-Link DNA ligase, as described above. The reaction mix was incubated with BspQ1 to digest the uncut pHel3-Myc vector and the reaction mix was then used for the transformation of *E. coli*. A plasmid containing the expected insert, designated as pKR66, was verified by restriction enzyme digestion and sequencing of the inserted DNA.

### 2.5. Transmission Electron Microscopy (TEM)

*H. pylori* strains were grown to late-log phase (OD_600_~1.0) in MHB supplemented with 5% heat-inactivated horse serum. Cells from 1 mL of culture were pelleted by centrifugation (550× *g*) and then resuspended in 125 μL of phosphate-buffered saline (PBS). Cells were fixed by adding 50 μL of 16% EM grade formaldehyde and 25 μL of 8% EM grade glutaraldehyde to the cell resuspension. Following an incubation at room temperature for 5 min, 10 μL of the cell suspension was applied to a 300 mesh, formvar-coated copper grid and incubated at room temperature for 5 min. The cell suspension was wicked off the grids using a filter paper, and the grids were washed 3 times with 10 μL of water. Cells were stained by applying 10 μL of 1% uranyl acetate to the grids for 30 s. After removing the stain with filter paper, the grids were washed three times with 10 μL of water and then air dried. Cells were visualized using a JEOL JEM 1011 transmission electron microscope. Reagents used for preparing cells for TEM were purchased from Thermo Fisher Scientific (Waltham, MA, USA).

### 2.6. Scanning Electron Microscopy (SEM)

*H. pylori* strains were grown, collected, and fixed as described for TEM preparation, and then resuspended in ~300 μL of PBS. The cell suspension was applied to poly-L-lysine-coated coverslips so as to nearly cover the coverslip, and then left overnight at room temperature. The following day, a secondary fixation step was performed using a 1:1:1 mixture of distilled water/4% osmium tetroxide/2× PBS for 1 h at 4 °C. Samples were washed once with PBS for 10 min, and then two washes with water were performed to remove excess salts before dehydration in an ethanol series that involved adding ethanol in 25% increments for 10 min at each step until reaching 100% ethanol. Samples were washed 3 times with 100% ethanol for 10 min each. Samples were then washed with a 1:1 mixture of hexamethyldisilazane (HMDS) and 100% ethanol for 15 min, followed by washes with 100% HMDS for 5 min and then 10 min. The coverslips were dried overnight at room temperature and then added to aluminum SEM stubs using adhesive circles. Samples were coated with gold by sputter coating in a vacuum chamber calibrated to 2 × 10^−1^ mbar/pa. Stubs were stored under desiccation. SEM was conducted using an FEI Tenio at 5.00 kV and a spot size of 7.0. Reagents used for SEM were purchased from Thermo Fisher Scientific (Waltham, MA, USA).

### 2.7. Fluorescence Microscopy

Cells of strain H93 (motile variant of *H. pylori* B128 Δ*hp0018* bearing plasmid pKR40, which carries the *hp0018*::*gfp* fusion) grown in BHI medium supplemented with heat-inactivated horse serum under microaerobic conditions to mid-logarithmic phase (OD_600_~1.0) were incubated with FM4-64 (5 uM final concentration) for 5 min at room temperature. Cells were harvested by centrifugation at 8700× *g* for 1 min, then washed three times by resuspending the cells and pelleting by centrifugation. A total of 20 μL of resuspended, stained cells was added to the slide, and after 10 min, the excess was wiped away. One drop of Prolong Gold antifade mountant (Thermo Fisher Scientific, Waltham, MA, USA) was added to the sample before placing a 1.5 mm coverslip on the cell sample. Cells were visualized with a Nikon Ti-U fluorescence microscope equipped with a Lumencor SOLA SM II light engine and fitted with a 100× oil immersion objective (NA 1.45) and GFP HISN Zero Shift and Texas Red Longpass filter sets. Image capture was performed with a CoolSNAP Myo camera (Teledyne Photometrics, Tucson, AZ, USA), controlled via the Nikon NIS-Elements BR software package (v. 4.20.01). Resultant images were processed with the ImageJ Fiji software package version 2.14.0/1.54f [29,30].

### 2.8. Preparation and Analysis of OMVs

Cells of *H. pylori* strains grown on TSA-HS medium were resuspended in 10 mL of filter-sterilized PBS and vortexed for 60 s to shear off the OMVs. Cells were removed from the suspension by centrifugation at 8700× *g* for 10 min and the resulting supernatant was passed through a 0.45 μm filter to remove any remaining cells. A sample of the filtrate was applied to formvar-coated copper grids and processed for TEM, as described above. OMVs were visualized using a JEOL JEM 1011 transmission electron microscope.

Proteins of *H. pylori* outer membrane vesicles (2 to 5 μg of total protein) were loaded onto an SDS–polyacrylamide gel and subjected to electrophoresis until the proteins entered the resolving gel. Gel slices containing the proteins were removed and sent to the University of Georgia Proteomics and Mass Spectrometry facility for protein identification. Proteins in the gel slices were digested with trypsin and the resulting peptides were analyzed by LC-MS/MS with a ThermoScientific Orbitrap Velo Elite mass spectrometer coupled with nano-HPLC using a 90 min elution gradient. Mass spectrometry data were searched against a protein database using the Mascot Server 2.7 software search engine (Matrix Science, Columbus, OH, USA) for protein identification.

### 2.9. DNA Sequencing and Analysis

gDNA from the *H. pylori* strains was purified using the Wizard genomic DNA purification kit (Promega) and submitted to the SeqCenter (Pittsburgh, PA, USA) for genomic library preparation and Illumina sequencing. Reads for the *H. pylori* gDNA sequence were mapped using the *breseq* computational pipeline [31] with the published NCBI genome for *H. pylori* B128 (Accession no.: NZ_CP024951.1).

### 2.10. Co-Immunoprecipitation Assay

Wild-type *H. pylori* B128 and the *H. pylori* strain expressing the HP0018-myc fusion protein (strain H141) were grown on TSA-HS plates, harvested, and resuspended in 7 mL of phosphate-buffered saline (PBS) with Pierce Protease Inhibitor (Thermo Fisher Scientific, Waltham, MA, USA). Cells were lysed at 18,000 psi with a French press and the resulting cell lysate was centrifuged at 7700× *g* for 10 min to remove unlysed cells and cell debris. The resulting supernatant was centrifuged at 100,000× *g* to pellet membrane vesicles, which were subsequently resuspended in PBS buffer containing 50 mM n-dodecyl-β-D-maltoside (DDM) (Chem-Impex International, Wood Dale, IL, USA). The solution was diluted to 20 mM DDM, the insoluble material was pelleted by centrifugation at 10,000× *g* for 10 min, and the resulting supernatant was used for the co-IP procedure using the Pierce c-Myc Tag Magnetic IP/Co-IP kit (Thermo Fisher Scientific), as described by the supplier. Samples were incubated with the magnetic beads either overnight at 4 °C or at room temperature for 30 min. Magnetic beads with bound proteins were washed at least 10 times with a 1:20 solution of Buffer 2 (supplied with the kit) containing 20 mM DDM. Proteins were eluted from the magnetic beads using 100 μL of 1× non-reducing sample buffer (supplied with the kit) followed by an incubation at 95–100 °C for 5 to 10 min. Samples were loaded on a 12% SDS-PAGE gel and subjected to electrophoresis until the samples entered the top of the resolving gel. The protein band was excised from the gel and submitted to the University of Georgia Proteomics and Mass Spectrometry Facility for analysis by in-gel trypsin digestion followed by LC-MS/MS on the Orbitrap mass spectrometer coupled with nano-HPLC using a 90 min elution gradient.

### 2.11. In Silico Modeling of Protein–Protein Interactions

Predicted tertiary structures of HP0018, HP0772 (AmiA), and HP1572 (MltD) were generated by AlphaFold [32]. AlphaFold2 multimer version 2.3.1 in UCSF ChimeraX 1.5 [32] was used to model interactions of the predicted structures using the University of Georgia Sapelo2 high performance computing cluster. For each pair of proteins, 25 ranked models were generated. PISA (Proteins, Interfaces, Structures, and Assemblies; https://www.ebi.ac.uk/pdbe/pisa/ (accessed on 19 August 2024), an interactive tool for examining protein–protein interfaces and predicting quaternary structures, was used to evaluate the energetic likelihood of predicted interactions for each model. PISA provides information on protein–protein interfaces, including the interface area, solvation free energy gain upon formation of the interface (Δ^i^G), and the P-value of the solvent free energy gain or interface specificity (Δ^i^G *p*-value), and displays all interfacing residues and any bonds that they form. A negative Δ^i^G value corresponds to hydrophobic interfaces or positive protein affinity. The *p*-value is a measure of interface specificity that measures the probability of obtaining a lower than observed Δ^i^G when the interface atoms are picked randomly from the protein surface. A *p*-value that is <0.5 indicates an interface where the hydrophobicity is greater than would be the average for a given structure, and the interface surface is thus potentially interaction-specific. In addition to Δ^i^G *p*-values < 0.5, calculated values that correspond to energetically favorable protein interfaces include an interface area > 1000 Å^2^ and a Δ^i^G < 0 kcal/mol.

## 3. Results

### 3.1. Deletion of hp0018 in H. pylori B128 Results in an Unusual Hypervesiculation Phenotype

HP0018 is a predicted lipoprotein of unknown function that is prevalent in FS^+^ *Helicobacter* species but is absent or underrepresented in FS^−^ *Helicobacter* species [21]. The HP0018 prolipoprotein is 469 amino acids in length and is encoded in an operon by itself [33]. HP0018 homologs are widespread among *H. pylori* strains, as a blastp search of the Joint Genome Institute’s Integrated Microbial Genomes and Microbiomes database (https://img.jgi.doe.gov/ (accessed on 12 March 2024) using the Homolog Display feature of the website identified HP0018 homologs with high confidence (bit scores > 824, E-values = 0.0, homology across the full-length of the protein) in 766 *H. pylori* genome sequences. Little is known about flagellar sheath biogenesis, and given that, as a lipoprotein, HP0018 likely localizes to the periplasm, it was a good candidate for having a role in sheath formation. To investigate the function of HP0018, we introduced an unmarked deletion of the *hp0018* homolog in *H. pylori* B128 (CV725_07715) and characterized the resulting mutant (designated as strain H19).

**Figure 1:**
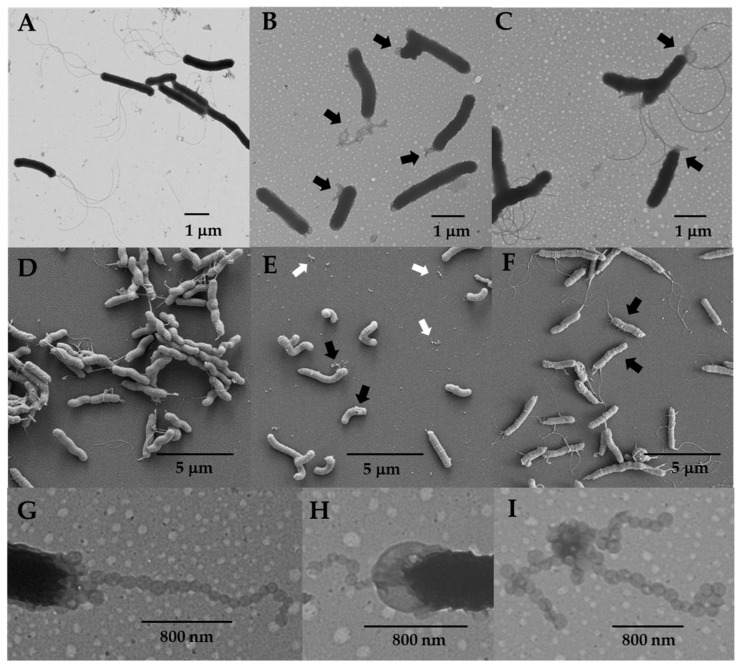
TEM and SEM images of wild-type *H. pylori* B128, the non-motile Δ*hp0018* mutant (H19), and the motile Δ*hp0018* isolate (H23). (**A**,**D**) TEM and SEM images, respectively, of wild-type *H. pylori* B128 cells. Note that the cells in the panel are flagellated. (**B**,**E**) TEM and SEM images, respectively, of H19. Note the chains of OMVs near the cell poles for many of the cells, which are indicated by the black arrows. Material that we infer to be free OMVs are indicated by the white arrows in (**E**). (**C**,**F**) TEM and SEM images, respectively, of strain H23. Note that the majority of the cells are flagellated and some of the cells appear to have OMVs on the cell surface (black arrows). (**G**–**I**) Close-up TEM images of chains of OMVs attached to cells (**G**,**H**) or detached from cells (**I**). Images shown in (**G**–**I**) are of strain H19. The magnification for the SEM images was 15,000×.

The motility of strain H19 was assessed by inoculating it into soft agar medium and allowing the cells to swim from the point of inoculation and multiply to produce a zone of growth or swim halo. Strain H19 produced no swim halo or a very small swim halo compared to the wild-type when inoculated into soft agar medium. Cell morphologies of H19 and wild-type *H. pylori* B128 were examined by TEM and SEM using cells from cultures grown in liquid medium to mid-logarithmic phase. Most of the H19 cells were aflagellated (Figure 1B,E), although flagellated cells were occasionally seen. The H19 cells produced abundant amounts of OMVs (Figure 1B). Examining over 200 H19 cells from three biological replicates by TEM, ~90% of the cells had OMVs associated with the cell surface. In contrast, <10% of the wild-type cells examined by TEM (*n* > 200; three biological replicates) had OMVs associated with the cell surface (Figure 1A). Interestingly, the H19 cells frequently produced chains of OMVs that tended to localize near the cell pole (Figure 1B,G,H). The OMV chains were unique to the Δ*hp0018* mutant, as we did not observe any such structures associated with wild-type cells. Detached chains of OMVs were often observed by TEM and SEM (Figure 1E,I), suggesting that the OMV chains were sheared readily from the cells as they were prepared for electron microscopy. The formation of OMVs is part of bacterial growth, and previous studies revealed that OMV formation in *H. pylori* increases as cultures enter the stationary phase [14,17]. Thus, the large amounts of OMVs produced by the H19 cells in logarithmic growth phase compared to wild-type cells was notable. Moreover, the chains of OMVs formed by the H19 cells seems to be a unique phenotype, as we are unaware of previous reports describing such morphological features in *H. pylori.* Another morphological distinction of the H19 cells was that they were less helical than wild-type cells, which was most evident in the SEM images (Figure 1D,E). 

### 3.2. HP0018 Is Not Required for Motility in Soft Agar Medium but Enhances Motility

**Figure 2:**
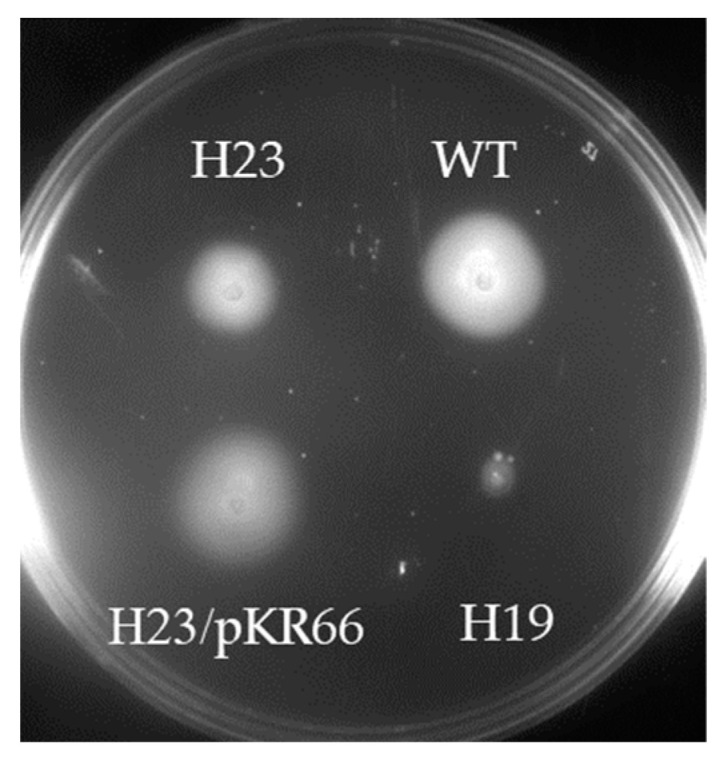
Motility of *H. pylori* strains in soft agar medium. *H. pylori* strains were stab inoculated into soft agar medium and incubated under microaerobic conditions for 7 d. Strains shown on the plate are wild-type *H. pylori* B128 (WT), H19 (non-motile Δ*hp0018* mutant), H23 (motile Δ*hp0018* isolate), and H23 carrying a derivative of plasmid pHel3 bearing a copy of *hp0018* (H23/pKR66).

To examine the basis of the motility defect of strain H19, motile variants of H19 were isolated by inoculating the strain into soft agar medium and incubating for 7 d to allow small swim halos to develop. Cells from the edge of the swim halo were used to inoculate fresh soft agar medium, which resulted in a larger swim halo. The process was repeated two more times, after which clonal isolates were obtained by streaking cells from the edge of the swim halo on TSA-HS medium. One of the motile isolates (designated as strain H23) was analyzed further. Strain H23 produced swim halos that were significantly larger than those formed by the parental H19 strain, but smaller than those generated by wild type (Figure 2). Introducing a copy of *hp0018* fused to a sequence encoding the c-myc epitope into strain H23 on the shuttle vector pHel3 (the plasmid bearing the *hp0018*-myc fusion was designated as pKR66) restored the motility of the strain in soft agar to the wild-type level (Figure 2). Swim halo formation results from cells migrating from the point of inoculation in soft agar medium, which involves both swimming and chemotaxis. It is unclear if the smaller swim halos generated by strain H23 are due to an impairment in swimming or chemotaxis. Cells of strain H23 bearing plasmid pKR66 did not form the large amounts of OMVs when grown to mid-logarithmic phase that were observed with the original strain H23 (Figure S1). Taken together, these results demonstrated that the HP0018-myc fusion suppressed the both the motility defect in soft agar medium and hypervesiculation phenotype of the Δ*hp0018* mutant.

**Figure 3:**
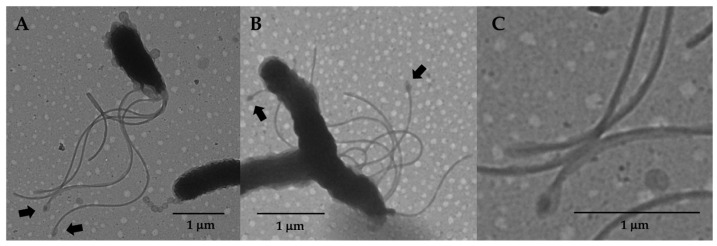
Flagella of strains H19 and H23. (**A**) TEM image of a flagellated cell and an aflagelleted cell of strain H19. Note that the flagellated cell has several OMVs on the cell surface, but lacks the chain of OMVs at the cell pole, while the aflagellated cell has a chain of OMVs at the cell pole. (**B**) TEM image of cells of strain H23. In both strains, note that some of the flagella had the characteristic bulb at the end of the filament (indicated by arrows). (**C**) Close-up image of flagella shown in panel A, where the bulb-like structures are clearly seen.

As expected, the H23 cells were well flagellated (Figure 1C,F). Similar to H19, the H23 cells were less helical than the wild-type cells (Figure 1D,F). Flagella of the H23 cells, as well as the occasional flagellated H19 cells, were sheathed and many had the characteristic bulb-like structure at their ends (Figure 3), indicating that HP0018 is not required for sheath formation. H23 cells typically had OMVs on the cell surface (Figure 1C), but the chains of OMVs were observed less frequently on H23 cells than they were on H19 cells. Similarly, the occasional flagellated H19 cell generally lacked the chains of OMVs (Figure 3A). Taken together, these observations suggest the OMV chains failed to form or were not stably associated with the cell when flagella were present.

Whole genome sequencing of H19 was performed to identify a mutation that might account for the aflagellation of the strain. Comparing the genome sequence of H19 with that of the parental, wild-type *H. pylori* B128 strain revealed several single nucleotide polymorphisms (SNPs) and indels (Table S2). Two of the mutations were in known flagellar genes, *motA* (encodes a motor stator protein) and *fliP* (encodes a component of the flagellar protein export apparatus). The SNP in *motA* was a missense mutation that changed Gly-201 to Asp and was present in 98.5% of the reads for that region of the genome. The mutation in *fliP* was a deletion of nucleotide 261, which is within a homopolymeric tract of eight cytidines. The deletion in the poly(C)-tract was present in about 90% of the reads for that region of the genome. Slipped-strand mispairing-mediated mutagenesis within the same poly(C)-tract in *fliP* was reported previously for *H. pylori* 26,695, and was proposed as a mechanism for switching between ‘on’ and ‘off’ phases for flagellation and motility [34]. It is not known if the phase variation of *fliP* is regulated and it may be stochastic. Since *H. pylori* motility is required for colonization in animal models [18,19], there must be a strong selective pressure for the motile phenotype in vivo. Conversely, it may be that following adherence to the gastric epithelium, the energy-saving loss of motility is advantageous for the bacterium.

Whole genome sequencing of H23 revealed that the deletion in the poly(C)-tract in *fliP* present in the parental H19 strain had reverted to the ‘on’ phase. The mutant *motA* allele identified in H19 was retained in H23, indicating that the MotA^G201D^ variant supported torque generation by the stator unit. Taken together, these observations indicate that a mutation in *fliP* was responsible for the aflagellated phenotype of H19. Moreover, these observations indicate that HP0018 is not required for flagellum assembly or motility.

An indel was noted in a third potential flagellar gene, CV725_07605 (HP0036 homolog), which encodes a predicted PflC homolog. The indel, a two-nucleotide deletion in a poly(T)-tract that introduced a frameshift mutation at codon 13, was present in both H19 and H23 (Table S2). PflC is predicted to form a motor accessory known as the medial disk in the *C. jejuni* flagellar motor [35], but this structure has not been identified in the *H. pylori* motor. If HP0036 does form a motor accessory in *H. pylori*, the frameshift mutation in the *hp0036* homolog in H23 suggests that the accessory is not required for motor function.

### 3.3. OMV Proteomes of Strains H19 and H23

**Figure 4:**
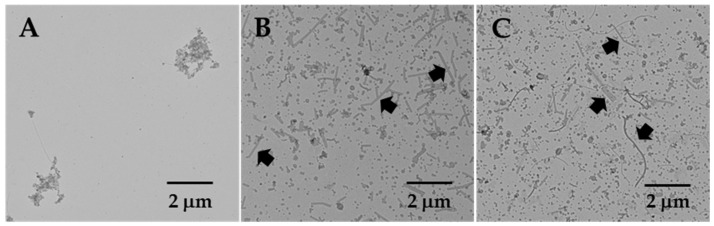
TEM images of OMVs isolated from wild-type, H19, and H23 strains. (**A**) OMV preparation from wild-type *H. pylori* B128. (**B**) OMV preparation from strain H19. (**C**) OMV preparation from strain H23. OMV preparations from H19 and H23 displayed tubular structures (indicated by arrows).

OMVs from H19 and H23 were isolated and compared with those prepared from wild-type *H. pylori* B128. OMVs were prepared by resuspending *H. pylori* cells in buffer, vortexing the cell suspensions to shear the OMVs from the cells, pelleting the cells by centrifugation, and removing any remaining cells by passing the supernatant liquids through a 0.45 μm filter. OMVs from the three *H. pylori* strains were initially examined by TEM (Figure 4). Consistent with the prevalence of OMVs on the H19 and H23 cells, these strains yielded denser concentrations of OMVs compared to the wild-type. In addition to the clusters of OMVs, tubular structures were observed in the samples prepared from H19 and H23 (Figure 4B,C). The tubular structures are not flagellar sheath fragments, since most of the H19 cells were aflagellated. Moreover, the tubular structures were not observed in the OMV sample prepared from wild-type cells.

Proteomes of the OMVs prepared from wild-type *H. pylori* B128, H19, and H23 were analyzed by mass spectroscopy. Each protein identified from the proteomic analysis was assigned a score calculated by the Mascot software package from the combined scores of all mass spectra that matched the amino acid sequence of the protein. While Mascot scores are not quantitative, scores generally correlate with the relative abundance of specific proteins. A total of 226 proteins were identified in the three samples. A complete list of identified proteins in the OMV samples is found in Table S3. A majority of the proteins identified in the OMV samples (~56%) were outer membrane proteins, predicted lipoproteins, periplasmic proteins, or flagellar proteins (Table S3). As expected, HP0018 was detected in the OMV sample from wild-type *H. pylori* B128 but not in the OMV samples from the two Δ*hp0018* strains. The protein content of the OMVs compared favorably with the proteomes of OMVs reported for two other *H. pylori* strains. Olofsson and co-workers identified 315 proteins in OMVs isolated from *H. pylori* CCUG17875 [14], while Zaran and co-workers identified 171 proteins that were significantly enriched in OMVs compared to the parental cell in *H. pylori* 26695 [17]. Eighty-four percent of the proteins that we identified in the *H. pylori* OMVs had been identified in one or both of the previous studies (Table S3).

With the exception of the absence of several flagellar proteins in the OMVs of H19, there were few qualitative differences observed in the protein content of the OMVs isolated from the three strains. Consistent with *fliP* being in the ‘off’ phase in H19, several flagellar proteins transported across the cell membrane by the flagellar type III secretion system were not detected in the OMVs of this strain. These proteins included FlaB (minor flagellin), FliD (filament cap protein), FlgE (hook protein), FlgL (hook-associated protein), FlgK (hook-associated protein), a FlgE homolog (HP0908), FlgD (hook cap protein), and FliK (hook length control protein) (Table S3). The major flagellin of the *H. pylori* flagellum, FlaA, was present in the OMVs of H19, although the Mascot score for FlaA in the sample was substantially lower than that of the other two samples (Table S3). The presence of FlaA in the OMVs of H19 was likely due to the small proportion of cells in the population where *fliP* was switched to the ‘on’ phase and allowed for flagellum assembly.

A couple of proteins with moderately high Mascot scores (>200) were identified in the OMVs of the two Δ*hp0018* strains that were not detected in the wild-type OMVs (Table S3). One of these proteins was ComH (HP1527; accession number: QDY55614.1), a periplasmic protein that is required for the natural transformation of *H. pylori* [36]. The other protein was a DUF1104 domain-containing protein (accession number: QDY56572.1) that corresponds to two open reading frames in *H. pylori* 26695 (HP0719 and HP0720). Both of these proteins were previously identified as being enriched in *H. pylori* 26695 OMVs [17], and so it seems unlikely that they have a role in the formation of the OMV chains since the researchers of this previous study did not report observing OMV chains. Two outer membrane proteins with moderately high Mascot scores (>330) were identified in the OMVs of H23 that were not detected in the OMVs of the other two strains. One of these proteins was FaaA (HP0609/HP0610), which is a VacA-like autotransporter that localizes to the flagellar sheath [37]. The other protein was HofB (HP1083), which is a predicted beta-barrel outer membrane protein. Taken together, there do not appear to be any proteins present in the OMVs of H19 or H23 that might account for the unusual tubular structures observed in Figure 4.

### 3.4. HP0018 Localizes to Specific Sites Within the Cell Envelope

**Figure 5:**
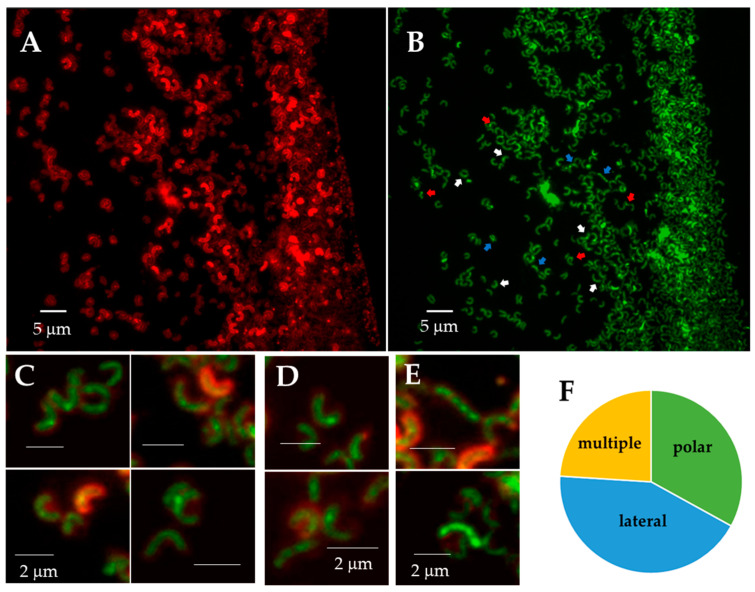
Localization of a HP0018-sfGFP fusion protein in *H. pylori* by fluorescent microscopy. (**A**,**B**) Identical X- and Y-planes and a slightly different Z-plane of a section of a slide with HP0018-sfGFP. (**A**) Red channel showing membrane staining with FM4-64, and (**B**) green channel showing the localization of HP0018-sfGFP. White arrows indicate the lateral localization of HP0018-sfGFP, red arrows a polar localization, and blue arrows multiple foci. (**C**–**E**) Merged channels of red (FM4-64) and green (HP0018-sfGFP) showing (**C**) lateral, (**D**) polar, and (**E**) multiple foci. For panels (**C**–**E**), all of the scale bars correspond to 2 µm. (**F**) The frequency of fluorescent foci at the cell pole (polar) and lateral sites was determined, as well as the frequency of cells with four or more fluorescent foci (multiple) (*n* = 133).

The localization of HP0018 in *H. pylori* was examined by fusing a superfolder green fluorescent protein (sfGFP) to the C-terminus of HP0018 and expressing the fusion protein in H23. Imaging the *H. pylori* cells expressing the HP0018-sfGFP by fluorescence microscopy revealed a strong tendency of the fusion protein to form fluorescent foci at the cell pole and lateral sites (Figure 5C–E). About 33% of the cells had fluorescent foci only at the cell pole, while ~43% of the cells had fluorescent foci only at lateral sites (i.e., non-polar sites) and ~24% of the cells had fluorescent foci at both the cell pole and lateral sites.

### 3.5. Identification of Potential HP0018 Interacting Partners

To investigate the possible physiological role of HP0018, we attempted to identify HP0018 interacting partners in a co-immunoprecipitation (co-IP) assay using the HP0018-myc fusion protein. The HP0018-myc fusion protein was immunoprecipitated from cell extracts using antibodies directed against the C-myc epitope, and proteins pulled down with the HP0018-myc fusion protein were identified by mass spectroscopy. Table 1 lists the proteins identified from the co-IP assay from one biological replicate, minus ribosomal proteins and ribosomal-associated proteins (e.g., elongation factor–Tu). As a negative control, co-IP assays were performed with three biological replicates of cell extracts prepared from wild-type *H. pylori* B128. For each protein listed in Table 1, the highest Mascot score from the three replicates of the negative control is indicated.

HP0018 was pulled down specifically in the co-IP assay, as it was detected in the cell extract containing the HP0018-myc fusion protein but not in any of the negative control replicates (Table 1). Four other proteins were detected in the co-IP assay with the sample containing the HP0018-myc fusion protein but not in the negative control replicates, suggesting that these proteins were also pulled down specifically. Two of these proteins, MurG and HP1294, are cytoplasmic proteins and are not likely to interact with HP0018 in vivo. The other two proteins, HP1235 and MltD (HP1572), are an integral membrane protein and periplasmic protein, respectively, and are therefore in locations in the cell where they could interact with HP0018 localized in the periplasm. HP1235 is a conserved protein of unknown function that belongs to the dolichyl-phosphate-mannose protein mannosyltransferase family (pfam13231). MltD is a lytic transglycosylase that is involved primarily in the rearrangement of the peptidoglycan layer [38].

Two other proteins, FlgS (HP0224) and AmiA (HP0772), were identified as potential HP0018 interacting partners, as they were underrepresented in the negative control replicates compared to the sample containing the HP0018-myc fusion protein (Table 1). Both of these proteins were detected in only one of the negative control replicates, and the Mascot scores for these proteins in the negative control were low. FlgS is a histidine kinase of a two-component system that is required for the transcription of the RpoN-dependent flagellar genes [39,40]. FlgS is a cytoplasmic protein, and so it is unlikely that it interacts with HP0018 in vivo. AmiA is a peptidoglycan hydrolase that is required for the morphological transition of *H. pylori* cells from spiral to coccoid form [41]. Since AmiA is a periplasmic enzyme, it is in a location in the cell where it could interact with HP0018. Although Chaput and co-workers annotated HP0772 as AmiA [41], the protein is more similar structurally to AmiB and AmiC, two other N-acetylmuramoyl-L-alanine amidases in *E. coli*. Specifically, *E. coli* AmiC has an N-terminal, peptidoglycan-binding domain known as an AMIN domain, which consists of a β-sandwich of two symmetrical four-stranded β-sheets [42]. Tertiary structures of *E. coli* AmiB and HP0772 predicted by AlphaFold indicate that both proteins have putative AMIN domains at their N-termini, whereas *E. coli* AmiA lacks this domain. We will continue to refer to HP0772 as AmiA though to avoid confusion.

**Figure 6:**
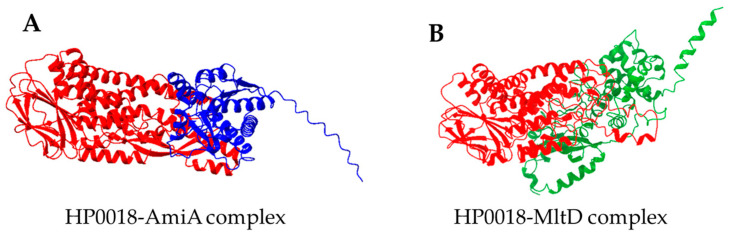
AlphaFold2 predictions of protein–protein interactions between HP0018 and AmiA or MltD. (**A**) Most energetically favorable model for interactions between HP0018 (shown in red) and the catalytic domain of AmiA (shown in blue). (**B**) Most energetically favorable model for interactions between HP0018 (shown in red) and MltD (shown in green).

Interactions of HP0018 with AmiA and MltD were modeled using AlphaFold2 multimer to obtain supporting evidence for interactions between these proteins. Twenty-five models were examined for each protein pair. In addition to the full-length AmiA, interactions of HP0018 with only the catalytic domain of AmiA were modeled. The energetic likelihood of predicted interactions for each model was evaluated using PISA. Models for interactions between HP0018 and AmiA or MltD predicted by PISA to be the most energetically favorable are presented in Figure 6. In the modeled interactions between HP0018 and the AmiA catalytic domain (Figure 6A), PISA values for the interface area, Δ^i^G, and Δ^i^G P-value were 1374.8 Å^2^, −19.4 kcal/mol, and 0.034, respectively. The HP0018-AmiA interface also included six hydrogen bonds and one salt bridge. In the modeled interactions between HP0018 and MltD (Figure 6B), PISA values for the interface area, Δ^i^G, and Δ^i^G P-value were 6238.6 Å^2^, −49.6 kcal/mol, and 0.301, respectively. The HP0018-MltD interface also included 94 hydrogen bonds and 11 salt bridges. Both of the modeled interfaces are predicted by PISA to be energetically favorable, indicating the feasibility of the interactions of HP0018 with AmiA and MltD that were predicted from the co-IP assays.

## 4. Discussion

Flagellar sheath biogenesis in bacteria is a poorly understood process. HP0018 was identified previously as a protein that is present in FS^+^ *Helicobacter* species but is absent in FS^−^ *Helicobacter* species [21], and we reasoned that it may have a role in sheath formation. To address this hypothesis, we deleted *hp0018* in *H. pylori* B128 and characterized the phenotype of the resulting mutant. Although the loss of HP0018 in *H. pylori* B128 did not appear to impact sheath formation (Figure 2), it did result in increased formation of OMVs that frequently manifested as long chains of OMVs at or near the cell pole in aflagellated cells of strain H19 (Figure 1). It was difficult to assess the proportion of H19 cells that had OMV chains since the structures were sheared readily from the cells while preparing the cells for electron microscopy. Nevertheless, cells with OMV chains were easily observed, which suggested that a high proportion of the cells possessed the OMV chains. It will be important to confirm that the hypervesiculation phenotype associated with the loss of HP0018 in *H. pylori* B128 is not strain specific by deleting *hp0018* in other *H. pylori* strains and examining the phenotypes of the resulting mutants.

OMVs are formed as a part of normal bacterial growth [10]. Previous studies showed that OMV formation by *H. pylori* cells increases as cultures enter the stationary phase [14,17]. Olofsson and co-workers reported that *H. pylori* strain CCUG17875 grown on Brucella blood agar medium produced small numbers of OMVs during logarithmic growth, but that OMV production increased upon entry into the stationary phase, and cells harvested during the late stationary phase produced large amounts of OMVs [14]. Zavan and co-workers reported the same trend of increased OMV production in *H. pylori* 26695 as cultures progressed from the early to late logarithmic phase and then into the stationary phase [17]. While OMV production is a normal part of *H. pylori* growth, the OMV chains observed for the aflagellated cells of strain H19 seems to be a unique phenotype, as we are unaware of previous reports describing such morphological features in *H. pylori.*

The chains of OMV were observed infrequently on the flagellated cells of strain H19 where *fliP* had switched to the ‘on’ phase. A couple of reasons may account for the apparent mutual exclusivity of the OMV chains and flagella in the Δ*hp0018* mutant (i.e., strain H23 or the occasional cell of strain H19 that was flagellated). Firstly, formation of the OMV chains may have been prevented by the presence of the flagella at the cell pole. For example, the flagellar sheath may affect the surrounding outer membrane region so as to prevent the OMV chains from forming. Alternatively, the *H. pylori* flagellar motor has several accessories that are not present in the archetypal motors of *Escherichia coli* and *Salmonella enterica* [43,44]. Some of the *H. pylori* motor accessories are associated with the outer membrane and may have prevented formation of the OMV chains in strain H23 or the flagellated cells of strain H19. A second possible explanation is that the OMV chains were sheared off by rotation of the flagella. Alternatively, HP0018 may have a role in coupling sheath biogenesis with flagellum assembly, and the OMV chains may have resulted from sheath formation initiating prematurely in the absence HP0018.

Although FS^−^ *Helicobacter* species lack HP0018 homologs, *C. jejuni* has sheath-less flagella but possesses a homolog of the HP0018 homolog, Cj0089. Cj0089 is encoded in an operon with two other predicted lipoproteins, Cj0090 and Cj0091. Oakland and co-workers disrupted *cj0089* and *cj0091* in *C. jejuni* NCTC 11168 and characterized phenotypes related to host colonization for the resulting mutants [45]. The *C. jejuni cj0091* mutant, but not the *cj0089* mutant, displayed significant reductions in both adherence to human intestinal cell line INT 407 cells and colonization of the chick cecum [45]. The researchers did not report on the cellular morphology of the *C. jejuni cj0089* mutant, and so it is not known if disrupting *cj0089* resulted in hypervesiculation.

Since genes within an operon often have roles in a common function, it is worthwhile to consider the synteny between *hp0018* homologs and other genes. Although *hp0018* appears to be in an operon by itself in *H. pylori*, in many enterohepatic *Helicobacter* species, the *hp0018* homolog is within a predicted operon with a *hp1457* homolog and immediately upstream of this gene. HP1457 is a homolog of LpoB, a lipoprotein that is required for the activation of the penicillin-binding protein PBP1B [46]. PBP1B is a bifunctional peptidoglycan synthase that has both glycan chain-polymerizing and peptide-crosslinking activities [47], and is part of the divisome complex that is responsible for the synthesis and splitting of the cell envelope at the division site [48]. HP1457 is an ortholog of *C. jejuni* Cj0091. The synteny between the *hp0018* and *hp1457* homologs in several *Helicobacter* species suggests these genes may have roles in a similar biological process. Given that *H. pylori* HP1457 is a LpoB homolog that may regulate the activity of PBP1B, by extension, HP0018 may have a similar role in remodeling the peptidoglycan layer. Consistent with this idea was the identification of the peptidoglycan hydrolases MltD and AmiA as potential HP0018 interacting partners based on the co-IP assays with the HP0018-myc fusion protein (Table 1). The interactions of HP0018 with MltD and AmiA were supported using AlphaFold to model complexes between these proteins along with PISA to calculate the energetic likelihood of protein interfaces in the models (Figure 6). Future experiments to confirm that HP0018 interacts with MltD and AmiA, however, are needed to clarify the role of HP0018 in *H. pylori*. 

The murein sacculus maintains the shape of the cell in bacteria, and several peptidoglycan hydrolases have been identified as having roles in determining the helical shape of *H. pylori* cells [49,50,51,52]. MltD and AmiA have not been shown to have roles in cell shape determination in *H. pylori*, but given their enzymatic activities, it is not unreasonable to postulate that they play a role in remodeling the murein sacculus. HP0018 may regulate the activity of MltD and AmiA, and the unregulated activity of these enzymes in the absence of HP0018 may have been responsible for the decreased helical shape of strains H19 and H23 (Figure 1D–F). Moreover, the slight motility defect of strain H23 in soft agar medium may be due to the altered cell shape of the mutant, as *H. pylori* mutants with a straight-rod morphology have motility defects in viscous environments [53]. If the absence of HP0018 does indeed result in the unregulated activities of MltD or AmiA, a relevant question is how does this relate to the hypervesiculation phenotype of the Δ*hp0018* mutant? The biogenesis mechanisms for bacterial OMV formation are poorly understood, although several mechanisms have been proposed [10]. One proposed mechanism of OMV production posits that the local accumulation of peptidoglycan fragments or misfolded proteins in the periplasm induced the curvature of the outer membrane to initiate OMV formation [10]. Such a mechanism could potentially explain a connection between the unregulated activities of MltD or AmiA and the unusual hypervesiculation phenotype of strain H19. Further investigations into MltD and AmiA and whether HP0018 modulates the activities of these enzymes will likely provide valuable information on cell shape determination in *H. pylori*, and may provide clues on the molecular basis for the unusual hypervesiculation of strain H19.

## 5. Conclusions

*H. pylori* HP0018 is a predicted lipoprotein that is conserved in many *Helicobacter* species. Cells of a *H. pylori* B128 Δ*hp0018* mutant were less helical in shape compared to wild-type cells, and also produced large amounts of OMVs that frequently formed long chains near the cell pole. Co-IP assays with HP0018 identified two enzymes involved in modification of the cell wall peptidoglycan, AmiA and MltD, as potential HP0018 interacting partners. We hypothesize that HP0018 regulates the activity of AmiA and/or MltD, and in the absence of HP0018, the unregulated activity of these enzymes affects the peptidoglycan layer so as to alter the cell shape and stimulate the formation of OMVs.

## Figures and Tables

**Table 1:** Proteins identified from the co-IP assay with the HP0018-myc-tagged protein.

		HP0018-myc Sample		WT (Negative Control)	
Accession	Description	^1^ Mascot Score	^2^ Peptides	^1^ Mascot Score	^2^ Peptides
QDY56472.1	IMP dehydrogenase GuaB, HP0829	1644	25	1659	25
QDY56231.1	RNA helicase RhpA, HP0247	1504	23	316	10
QDY55932.1	hypothetical protein, HP1124	923	17	118	3
QDY56010.1	mechanosensitive ion channel family protein, HP0415	823	19	316	9
**QDY56228.1**	**two-component sensor histidine kinase FlgS, HP0244**	**748**	**16**	**74**	**1**
QDY56734.1	sel1 repeat family protein, HP0519	703	11	561	14
QDY56639.1	outer inflammatory protein OipA, HP0638	475	7	117	6
**QDY56525.1**	**N-acetylmuramoyl-L-alanine amidase AmiA, HP0772**	**461**	**13**	**70**	**2**
QDY56975.1	pyridoxine 5′-phosphate synthase PdxJ, HP1582	437	9	201	5
QDY56071.1	urease subunit alpha UreA, HP0073	416	8	510	9
**QDY56955.1**	**HP0018**	**372**	**8**	**nd**	**nd**
QDY56262.1	mechanosensitive ion channel family protein, HP0284	356	7	104	3
**QDY55904.1**	**undecaprenyldiphospho-muramoylpentapeptide beta-N-acetylglucosaminyltransferase MurG, HP1155**	**288**	**5**	**nd**	**nd**
QDY56151.1	Sel1-like repeat protein HcpD, HP0160	252	9	509	9
**QDY56984.1**	**lytic transglycosylase MltD, HP1572**	**218**	**7**	**nd**	**nd**
QDY56970.1	DUF3944 domain-containing protein, HP1588	216	5	527	11
QDY56963.1	molecular chaperone GroEL, HP0010	174	8	1487	31
QDY56571.1	DUF1104 domain-containing protein, HP0721	169	2	116	1
**QDY55828.1**	**hypothetical protein, HP1235**	**169**	**5**	**nd**	**nd**
QDY56232.1	hypothetical protein, HP0248	130	3	525	13
QDY55919.1	chromosome partitioning protein ParB, HP1138	121	4	264	9
QDY56153.1	porphobilinogen synthase HemB, HP0163	117	4	70	2
**QDY55781.1**	**glycosyltransferase family protein, HP1284**	**102**	**5**	**nd**	**nd**

^1^ Indicates Mascot scores for proteins that were identified in the co-IP samples prepared with cell extracts from strain H23 expressing the HP0018-myc-tagged protein or wild-type *H. pylori* B128 (WT). Proteins discussed in the text are highlighted in bold ^2^ Indicates the number of peptide fragments generated following trypsin digestion that were identified for each protein. nd—not detected.

## Data Availability

The original contributions presented in the study are included in the article and Supplementary Materials; further inquiries can be directed to the corresponding author.

## Supplementary Materials

The following supporting information can be downloaded at https://www.mdpi.com/article/10.3390/cells13171438/s1, Table S1. Primers, plasmids, and strains generated in the study; Table S2. Mutations identified in the original *H. pylori* Δ*hp0018* mutant (strain H19) and a motile variant of the Δ*hp0018* mutant (strain H23) compared to wild-type *H. pylori* B128; Table S3. Proteins identified in OMV samples from wild-type *H. pylori* B128, the non-motile Δ*hp0018* mutant (strain H19), and the motile Δ*hp0018* mutant (strain H23); and Figure S1. TEM images of the motile *H. pylori* Δ*hp0018* mutant complemented with a plasmid expressing the HP0018-myc fusion protein.
