# Supplementary material for: Helicobacter pylori HP0018 Has a Potential Role in the Maintenance of the Cell Envelope
Source: Cells. 2024 Aug 27;13(17):1438. doi: 10.3390/cells13171438 (PMC11394524; doi:10.3390/cells13171438)
Supplement: Supplementary file 1 [file cells-13-01438-s001.zip › Figure S1.pdf]

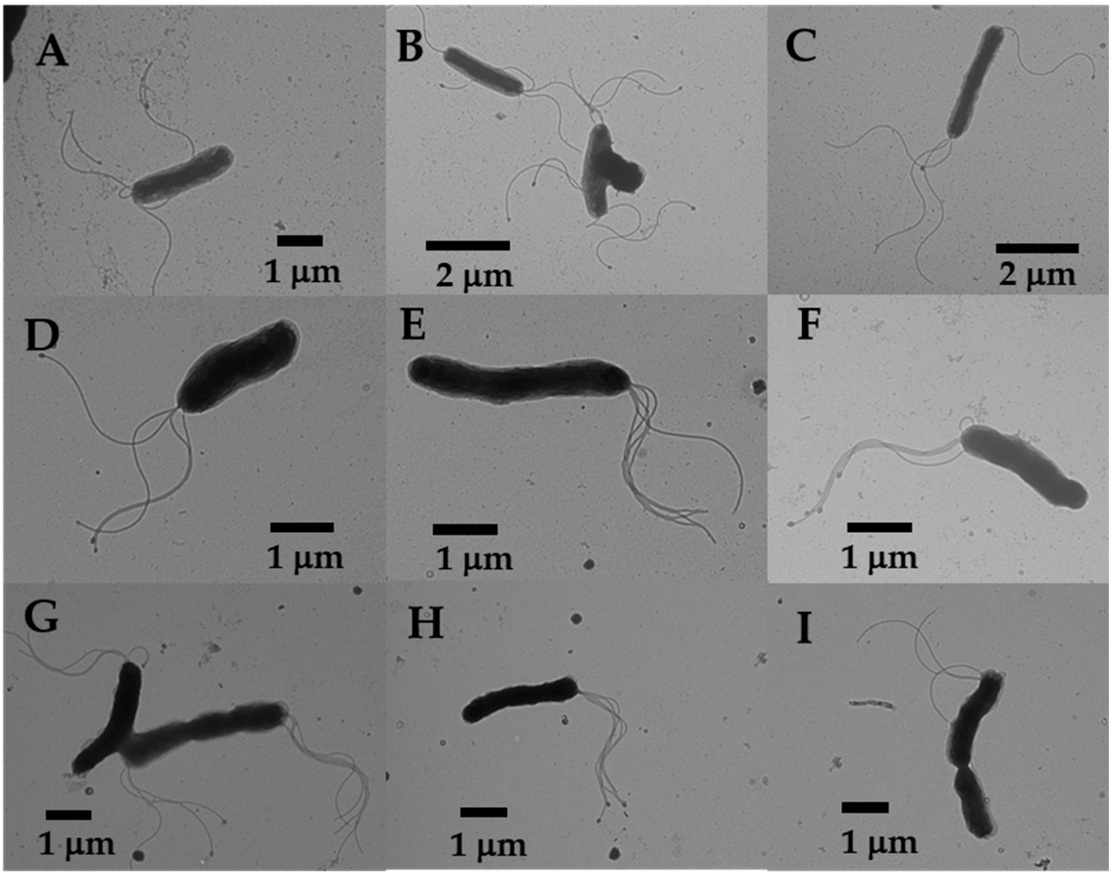

Figure S1. TEM images of the motile *H. pylori* Δ*hp0018* mutant complemented with plasmid expressing the HP0018-myc fusion protein. Examples of wild-type *H. pylori* B128 cells are shown in panels A-C, while examples of the *H. pylori* Δ*hp0018* mutant bearing the plasmid encoding the HP0018-myc fusion protein are shown in panels D-I.
