# Supplementary material for: Helicobacter pylori HP0018 Has a Potential Role in the Maintenance of the Cell Envelope
Source: Cells. 2024 Aug 27;13(17):1438. doi: 10.3390/cells13171438 (PMC11394524; doi:10.3390/cells13171438)
Supplement: Supplementary file 1 [file cells-13-01438-s001.zip › Table S1_strains, plasmids, and primers.pdf]

Table S1. Strains, plasmids, and primers generated for the study.

H. pylori **strains**	
**name**	**relevant genotype**
H13	*H. pylori* B128 *hp0018::k*an-*sacB*
H19	*H. pylori* B128 *Δhp0018 fliP261* (deletion of nucleotide 261 in *fliP*)
H23	*H. pylori* B128 Δ*hp0018* (wild-type *fliP* allele)
H93	H23 bearing plasmid pKR40
H141	H23 bearing plasmid pKR66
**plasmids**	
**name**	**description**
pRK12	pGEM-T Easy vector containing *hp0018* flanking regions; Ap^R
pRK14	pRK12 derivative with kan^R-*sacB* cassette; Ap^R, Kn^R
pRK31	pGEM-T Easy vector containing *hp0018-gfp* fusion; Ap^R
pKR40	pHel3 containing *hp0018-gfp* fusion; Kn^R
pHel3-myc	Modified pHel3 w *fliF* promoter, c-myc tag, tandem BspQI cloning sites; Kn^R
pKR66	pHel3-myc derivative that expresses HP0018-myc fusion protein; Kn^R
**primers**	
**name**	**sequence**
Primer 51	5′-AAACACAATTTCAATGTGCCTTTGA-3′
Primer 52	5′-GAATTCGATTATCCTCGAGTAAAATTACCGACATCAACAGAACG-3′
Primer 53	5′-GATAATCGAATTCGCTAGCTTTTAAAAACGGGTTTGTTTTGAGT-3′
Primer 54	5′-AAAATCCTATCCGCTTCATAGATCC-3′
Primer 102	5′-GCTAGCCTTTTTTAGAAAACACCCCTTTGTA-3′
Primer 103	5′-CATGCTACCGCCACCACCTTTTAAATGACTCAAAACAAACCCG-3′
Primer 104	5′-GGTGGTGGCGGTAGCATGAGCAAAG-3′
Primer 105	5′-GGATCCTTATTTATACAATTCATCCATGCCA-3′
Primer 206	5′-AAGCTCTTCCATGAAAATATTCGTTCTGTTGATGT-3′
Primer 207	5′-AAGCTCTTCAACCTTTTAAATGACTCAAAACAAACCC-3′
