# Supplementary material for: Helicobacter pylori HP0018 Has a Potential Role in the Maintenance of the Cell Envelope
Source: Cells. 2024 Aug 27;13(17):1438. doi: 10.3390/cells13171438 (PMC11394524; doi:10.3390/cells13171438)
Supplement: Supplementary file 1 [file cells-13-01438-s001.zip › Table S2_SNPs and indels.pdf]

Table S2. Mutations identified in the original *H. pylori* Δ*hp0018* mutant (strain H19) and a motile variant of the Δ*hp0018* mutant (strain H23) compared to wild-type *H. pylori* B128.

**Strain H19**				
**NCBI designation**	**Gene/description**	**Mutation**	[1]**Impact**	[2]**Frequency**
CV725_RS01525	sugar MFS transporter	ACA→ATA	Thr83Ile	98.7%
CV725_RS02980	DASS family sodium-coupled anion symporter	AGG→GGG	Arg87Gly	98.7%
CV725_RS03080	HAMP domain-containing histidine kinase ArsS	$(C)_{11\rightarrow14}$ (1261/1281 nt)	Insertion of a Pro residue	82.4%
CV725_RS03820 *hugZ*	YbfB/YjiJ family MFS transporter/HugZ family heme oxygenase	$(T)_{15\rightarrow14}$ intergenic (-340/+172)	unknown	81.2%
CV725_RS04360	hypothetical protein	$(T)_{7\rightarrow6}$ (1040/1251 nt)	Leu347fs	98.0%
CV725_RS04600 *CV725_RS04605*	TonB-dependent receptor/catalase	$(A)_{5\rightarrow6}$ intergenic (-34/-290)	unknown	82.6%
motA	flagellar motor stator protein MotA	GGC→GAC	Gly201Asp	98.5%
fliP	flagellar type III secretion system protein FliP	$(G)_{8\rightarrow9}$ (254/747 nt)	Thr88fs	90.1%
CV725_RS06190	hypothetical protein	CCA→TCA	Pro78Ser	94.1%
CV725_RS06350	cag pathogenicity island protein	GTT→ATT	Val1058Ile	89.2%
CV725_RS06350	cag pathogenicity island protein	AAA→AGA	Lys921Arg	98.0%
CV725_RS06500	phospholipase A, pseudogene	$(G)_{10\rightarrow9}$ (683/1070 nt)	remains phase "off"	86.2%
babA *CV725_RS06645*	Hop family adhesin BabA/tRNA-Met	$(T)_{13\rightarrow12}$ intergenic (-131/+149)	unknown	87.9%
CV725_RS07515 *queF*	hypothetical protein/NADPH-dependent 7-cyano-7-deazaguanine reductase QueF	$(C)_{11\rightarrow10}$ intergenic (+11/+121)	unknown	92.5%
CV725_RS07605	PDZ domain-containing protein, PflC homolog	$(T)_{8\rightarrow6}$ (31-32/996 nt)	Leu13fs	99.2%
CV725_RS07695	HP0018 homolog	Δ1338 nt (37-1374/1410 nt)	deletion	98.0%
**Strain H23**				
**NCBI designation**	**Gene/description**	**Mutation**	**Impact**	**Frequency**
CV725_RS01525	sugar MFS transporter	ACA→ATA	Thr83Ile	98.1%
CV725_RS02390	rod shape-determining protein	GCG→GTG	Ala100Val	99.0%

CV725_RS02980	DASS family sodium-coupled anion symporter	AGG→GGG	Arg87Gly	98.9%
CV725_RS03820 *hugZ*	YbfB/YjiJ family MFS transporter/HugZ family heme oxygenase	$(T)_{15\rightarrow14}$ intergenic (-340/+172)	unknown	80.3%
CV725_RS04360	hypothetical protein	$(T)_{7\rightarrow6}$ (1040/1251 nt)	Leu347fs	98.1%
CV725_RS04600 *CV725_RS04605*	TonB-dependent receptor/catalase	$(A)_{5\rightarrow6}$ intergenic (-34/-290)	unknown	81.3%
motA	flagellar motor stator protein MotA	GGC→GAC	Gly201Asp	98.7%
CV725_RS05520	TIGR00366 family protein	$(T)_{7\rightarrow3}$ (810-813/1365 nt)	Phe271fs	96.5%
CV725_RS05620	SIR2 family protein, pseudogene	C→T (1752/2474 nt)	remains a pseudogene	98.1%
CV725_RS06350	cag pathogenicity island protein	GTT→ATT	Val1058Ile	90.2%
CV725_RS06350	cag pathogenicity island protein	AAA→AGA	Lys921Arg	98.7%
CV725_RS06500	phospholipase A, pseudogene	$(G)_{10\rightarrow9}$ (683/1070 nt)	remains phase "off"	84.3%
babA *CV725_RS06645*	Hop family adhesin BabA/tRNA-Met	$(T)_{13\rightarrow12}$ intergenic (-131/+149)	unknown	84.0%
hofG *CV725_RS06740*	outer membrane protein HofG/TonB-dependent receptor	$(A)_{14\rightarrow15}$ intergenic (-71/+258)	unknown	81.0%
CV725_RS07515 *queF*	hypothetical protein/NADPH-dependent 7-cyano-7-deazaguanine reductase QueF	$(C)_{11\rightarrow10}$ intergenic (+11/+121)	unknown	91.8%
CV725_RS07605	PDZ domain-containing protein, PflC homolog	$(T)_{8\rightarrow6}$ (31-32/996 nt)	Leu13fs	99.1%
CV725_RS07695	HP0018 homolog	Δ1338 nt (37-1374/1410 nt)	deletion	98.7%

[1]"fs" indicates frameshift

[2]Frequency is the number of times the mutation was identified relative to the total number of reads for that sequence.
