# Supplementary material for: Helicobacter pylori HP0018 Has a Potential Role in the Maintenance of the Cell Envelope
Source: Cells. 2024 Aug 27;13(17):1438. doi: 10.3390/cells13171438 (PMC11394524; doi:10.3390/cells13171438)
Supplement: Supplementary file 1 [file cells-13-01438-s001.zip › Table S3_OMV proteins.pdf]

Table S3. Proteins identified in OMV samples from wild-type *H. pylori* B128, non-motile Δ*hp0018* mutant (strain H19), and motile Δ*hp0018* isolate (strain H23).

		wild type		strain H19		strain H23		
accession	description	[1]Mascot score	[2]no. of peptides	[1]Mascot score	[2]no. of peptides	[1]Mascot score	[2]no. of peptides	[3]identified previously
WP_000726315.1	Hop family adhesin LabA, HP0025	8563.26	45	7857.20	47	14136.91	47	[1,2]
WP_000885488.1	flagellin A, HP0601	6436.44	30	583.07	2	12027.34	33	[1,2]
WP_145801814.1	Hop family adhesin BabA, HP1243	5197.13	31	6411.55	33	6276.06	34	[1,2]
WP_000751499.1	Hop family adhesin HopQ, HP1177	3089.26	28	3533.03	27	2858.25	27	[1,2]
WP_000010021.1	flagellin B, HP0115	2881.86	22	0.00	0	4904.40	33	[1,2]
EEC24754.1	flagellar filament cap protein FliD, HP0752	2838.63	38	0.00	0	4190.37	41	[1,2]
QDY56424.1	outer membrane beta-barrel protein HofC, HP0486	2792.37	22	4676.62	25	4547.96	24	[1,2]
QDY56215.1	disulfide isomerase, HP0231	2416.86	21	3017.51	22	2456.88	22	[1,2]
QDY56165.1	peptidylprolyl isomerase, HP0175	2160.94	28	2863.34	32	1642.09	24	[1]
WP_000795968.1	LPP20 family lipoprotein, HP1456	2125.95	18	2836.64	20	3052.54	22	[1,2]
WP_000595790.1	outer membrane beta-barrel protein HorE, HP0472	1759.30	9	821.63	6	1212.52	9	[1,2]
QDY55938.1	γ-glutamyltransferase, HP1118	1752.60	21	1955.03	22	1290.59	19	[1]
WP_001040308.1	chaperonin GroEL, HP0010	1711.37	24	1873.07	25	1461.94	20	[1]
EEC24951.1	predicted lipoprotein, HP0596	1709.44	14	2554.35	15	2861.28	17	[1,2]
WP_000720394.1	LPP20 family lipoprotein, HP1454	1662.02	24	1321.76	21	1030.46	23	[1,2]
WP_000738953.1	YceI family protein, HP0305	1645.09	12	1815.61	11	1807.77	12	[1,2]
QDY56091.1	predicted lipoprotein, HP0097	1613.61	10	1891.21	15	1839.18	14	[1,2]
WP_000646639.1	flagellar sheath lipoprotein HpaA, HP0797	1581.04	17	2260.23	18	1738.32	21	[1,2]
WP_000751159.1	outer membrane protein HopA, HP0229	1541.30	13	2132.45	16	1900.85	15	[1,2]
QDY56583.1	outer membrane protein HopE, HP0706	1475.83	14	1964.96	14	2033.19	15	[1,2]
QDY56399.1	secreted protease PqqE, HP1012	1462.35	19	1733.86	19	1182.48	18	[1,2]
QDY56619.1	secreted protease, HP0657	1440.76	19	1250.54	24	713.89	12	[1]
QDY56273.1	flagellar biosynthesis protein FlgL, HP0295	1306.95	27	0.00	0	2055.06	36	[1,2]
QDY56393.1	secreted protease, HP1019	1242.35	26	1397.28	24	1200.73	26	[1,2]
QDY56824.1	outer membrane protein HomA, HP0710	1185.88	19	2453.36	26	2371.84	27	[1,2]
WP_001020067.1	periplasmic binding protein, HP1564	1177.15	14	1547.95	16	1220.54	13	[1,2]

QDY55779.1	polyisoprenoid-binding protein, HP1286	1066.04	13	1419.28	12	745.10	10	[1,2]
QDY56435.1	flagellar hook protein FlgE, HP0870	1048.49	24	0.00	0	3441.91	35	[1,2]
WP_000715700.1	HpaA2 protein, HP0410	1021.95	11	1404.94	13	906.88	11	[1,2]
QDY55937.1	flagellar hook-associated protein FlgK, HP1119	996.79	20	0.00	0	1488.03	25	[1,2]
WP_192940664.1	outer inflammatory protein OipA, HP0638	958.47	10	1173.68	11	1246.73	11	[1,2]
EEC24262.1	ATP synthase subunit B, HP1132	947.10	13	1251.56	15	601.73	9	[1]
QDY56621.1	β-barrel assembly factor BamA, HP0655	944.64	18	1335.34	19	871.91	15	[1,2]
WP_000846456.1	DNA-binding protein Dps, HP0243	943.30	10	133.94	3	62.22	2	
QDY55857.1	elongation factor Tu, HP1205	901.38	17	584.96	14	321.00	6	[1]
WP_000731326.1	fibronectin type III domain-containing protein, HP0746	895.55	17	1133.22	18	1128.40	19	[1,2]
WP_001174690.1	thiol peroxidase Tpx, HP0390	886.03	9	647.37	9	439.82	5	
WP_000856042.1	periplasmic substrate-binding protein, HP1172	876.35	12	1153.04	16	798.92	13	[1]
QDY56995.1	periplasmic iron-binding protein, HP1561	876.07	10	1281.72	12	754.59	9	[1,2]
WP_000323697.1	DUF3944 domain-containing protein, HP1588	870.13	13	565.29	11	400.97	8	[1]
QDY56671.1	TolC family protein HefA, HP0605	853.46	13	1233.78	17	749.97	12	[1,2]
EEC24860.1	secreted protease, HP1350	850.79	24	1308.33	28	880.00	23	[1,2]
QDY56734.1	sel1 repeat family protein, HP0519	800.53	11	769.03	10	826.78	11	
QDY56121.1	hypothetical protein, HP0130	781.64	15	1258.04	16	1241.21	17	[1,2]
QDY56720.1	oncogenic effector CagA, HP0547	765.13	24	94.47	5	413.06	13	[1]
WP_001268551.1	SH3 domain-containing peptidase, HP0087	742.42	12	760.63	11	703.49	8	[1,2]
QDY55930.1	translocation protein TolB, HP1126	732.34	12	1017.56	20	601.22	12	[1,2]
EEC23889.1	outer membrane protein FecA-3, HP1400	716.90	18	1799.28	27	1513.75	22	[1,2]
QDY56118.1	outer membrane protein HorB, HP0127	715.16	7	907.78	8	639.32	5	[1,2]
EEC24215.1	hypothetical protein, HP0953	696.62	5	795.25	6	701.80	6	[1]
QDY56703.1	peptidase PepA, HP0570	688.04	11	0.00	0	163.12	4	[1]
QDY56994.1	periplasmic iron-binding protein, HP1562	686.36	11	1102.89	13	758.00	12	[1,2]
WP_000788701.1	outer membrane protein HopF, HP0252	682.15	13	887.03	13	725.36	12	[1]
QDY56993.1	alkyl hydroperoxide reductase TsaA, HP1563	675.46	7	1221.81	10	754.56	7	[1]
WP_000117378.1	citrate synthase, HP0026	649.33	9	408.33	7	240.07	4	[1]
QDY55923.1	F0F1 ATP synthase subunit alpha, HP1134	647.46	16	883.81	23	215.82	10	[1]

QDY56423.1	Catalase, HP0485	645.66	11	813.86	13	426.14	11	[1,2]
QDY56253.1	pentatricopeptide repeat domain, HP0275	644.50	15	844.91	13	364.50	8	[1,2]
QDY56801.1	flagellar hook protein FlgE2; HP0908	636.37	13	0.00	0	515.79	13	[1,2]
EEC25266.1	outer membrane protein HopG, HP0254	613.05	12	1068.26	12	1000.68	11	[1,2]
EEC25319.1	periplasmic solute-binding protein, HP0298	611.10	14	584.32	14	318.31	12	[1]
QDY56070.1	urease beta subunit UreB, HP0072	608.96	14	459.84	9	378.15	11	[1]
QDY56511.1	outer membrane beta-barrel protein HofF, HP0788	596.30	10	676.29	10	629.89	8	[1,2]
QDY56775.1	neuraminyllactose-binding hemagglutinin, HP0492	540.01	11	773.56	11	699.63	9	[1,2]
QDY57028.1	outer membrane protein HorL, HP1395	530.65	10	746.95	11	558.48	9	[1,2]
QDY56609.1	outer membrane protein HorF, HP0671	520.32	6	581.97	9	779.94	9	[1,2]
EEC24629.1	hypothetical protein, HP1285	504.58	7	674.83	7	695.08	8	[1,2]
EEC24785.1	aconitate hydratase, HP0779	503.83	10	317.44	5	262.19	3	
QDY56599.1	outer membrane protein FecA-1, HP0686	492.86	10	1121.51	17	698.57	8	[1,2]
QDY55893.1	outer membrane beta-barrel protein HofH, HP1167	485.71	12	960.24	13	996.76	14	[1,2]
WP_001206904.1	2-oxoglutarate synthase subunit alpha, HP0589	462.62	9	284.21	8	144.95	4	[1]
WP_000709627.1	DUF1104 domain-containing protein; HP0721	455.73	4	1369.84	4	555.40	4	[1,2]
QDY55647.1	TolC family protein, HP1489	442.74	8	536.37	9	480.77	7	[1,2]
QDY56645.1	hydrogenase large subunit, HP0632	439.21	12	775.65	13	513.34	11	[1]
EEC24175.1	predicted lipoprotein, HP1002	438.39	10	479.27	12	619.23	11	
EEC24379.1	outer membrane protein HopI, HP1156	432.62	7	892.82	10	689.63	6	[1,2]
QDY56985.1	septal ring lytic transglycosylase RlpA family protein, HP1571	412.73	7	762.97	10	500.30	8	[1,2]
QDY55887.1	hypothetical protein, HP1173	404.03	9	812.34	10	476.77	9	[1,2]
QDY55678.1	penicillin-binding protein activator LpoB, HP1457	399.90	5	953.29	8	987.80	10	[1,2]
WP_001228615.1	5'-nucleotidase C-terminal domain-containing protein, HP0104	392.90	12	413.54	10	264.99	7	[1,2]
WP_000915372.1	outer membrane protein HomD, HP1453	384.22	9	867.59	12	491.78	9	[1,2]
QDY56442.1	plasminogen-binding protein PgbB, HP0863	379.08	14	641.56	16	448.37	17	[1,2]
QDY55951.1	outer membrane protein HorH, HP1107	375.43	6	352.89	5	239.51	4	[2]
WP_000945748.1	TonB-dependent receptor FrpB-3, HP1512	373.56	9	1762.42	24	1451.37	20	[1,2]
QDY56476.1	thioredoxin-disulfide reductase TrxB, HP0825	372.73	6	183.92	5	143.76	5	[1]
QDY56296.1	periplasmic nuclease NucT, HP0323	368.53	5	593.40	9	407.79	6	[1,2]

QDY56126.1	predicted lipoprotein, HP0135	367.02	3	501.57	3	533.18	3	[2]
QDY55673.1	predicted lipoprotein, HP1463	355.58	5	382.58	6	340.37	7	[1,2]
WP_000467790.1	hypothetical protein, HP0599	351.46	7	415.37	6	409.07	8	[1]
QDY55940.1	Sel1-like repeat protein, HP1117	346.89	7	630.87	9	320.65	5	[1,2]
WP_001236618.1	flagellar motor accessory protein FlgP, HP0836	344.39	8	443.26	8	418.92	8	[2]
QDY56192.1	hypothetical protein, HP0204	334.15	5	349.01	4	109.01	2	[2]
QDY55739.1	copper resistance protein, HP1326	330.30	7	663.14	10	430.56	6	[2]
QDY55989.1	superoxide dismutase SodB, HP0389	320.70	7	384.95	10	78.97	2	[1]
WP_104932432.1	outer membrane protein HopK, HP0477	320.33	4	667.87	9	485.91	7	[1,2]
EEC24290.1	hypothetical protein, HP0973	319.10	5	600.28	9	346.03	6	[1]
WP_000033534.1	methyl-accepting chemotaxis protein, HP0099	314.00	9	193.75	4	0.00	0	[1]
EEC24268.1	peptidoglycan-associated lipoprotein PalA, HP1125	308.06	5	488.58	7	618.79	7	[1]
WP_000532521.1	outer membrane beta-barrel protein, HP0726	300.80	6	560.28	8	510.68	6	[1,2]
QDY56219.1	Sel1 repeat protein HcpE, HP0235	298.87	5	437.95	7	323.40	5	[1,2]
QDY56617.1	periplasmic chaperone SurA, HP0659	296.67	7	557.54	10	401.30	9	[1,2]
QDY56947.1	isocitrate dehydrogenase Icd, HP0027	296.66	8	244.73	9	93.43	5	[1]
WP_000945142.1	plasminogen-binding protein PbgA, HP0508	295.92	7	348.13	6	279.35	5	[1,2]
QDY56799.1	flagellar hook-length control protein FliK, HP0906	291.45	6	0.00	0	635.27	12	[2]
WP_000591329.1	outer membrane protein HopL, HP1157	291.19	7	398.61	9	456.27	9	[1]
QDY56071.1	urease subunit alpha UreA, HP0073	276.58	8	908.39	5	253.14	2	[1]
WP_000061442.1	FAD-dependent oxidoreductase, HP0086	274.41	5	114.04	4	81.71	3	[1]
QDY56597.1	acetyl-CoA C-acetyltransferase FadA, HP0690	274.17	5	142.94	2	0.00	0	[1]
WP_001215737.1	aliphatic amidase, HP0294	272.17	6	120.60	2	178.64	4	[1]
QDY56800.1	flagellar hook cap protein FlgD, HP0907	270.62	7	0.00	0	205.82	4	[1,2]
QDY56464.1	outer membrane protein, HP0839	256.72	4	303.81	6	325.92	4	[1,2]
EEC24940.1	membrane fusion protein, HP0606	250.31	7	446.78	7	158.04	4	[1]
QDY55668.1	outer membrane protein HorJ, HP1469	233.90	6	361.48	7	266.69	6	[1,2]
QDY55617.1	predicted lipoprotein, HP1524	228.57	5	449.23	7	350.36	5	[2]
EEC24201.1	periplasmic solute-binding protein, HP0940	224.83	7	163.92	7	221.40	5	[1,2]
WP_000866621.1	peptide-methionine (R)-S-oxide reductase MsrAB, HP0224	208.84	3	734.23	13	223.35	4	[1,2]

WP_000492196.1	thio:disulfide exchange protein DsbC, HP0377	206.42	4	325.52	6	254.90	2	[1]
QDY55873.1	carbonic anhydrase, HP1186	202.95	5	268.57	5	205.07	6	[1,2]
QDY56593.1	hypothetical protein, HP0694	197.09	3	417.59	5	161.88	3	[1,2]
QDY55932.1	hypothetical protein, HP1124	196.46	4	361.76	10	191.52	7	[1,2]
WP_001861350.1	META domain-containing protein, HP1462	195.37	4	437.17	5	340.21	6	[1,2]
QDY55749.1	50S ribosomal protein L2, HP1316	191.52	4	97.63	2	0.00	0	[1]
QDY55896.1	NAD(P)/FAD-dependent oxidoreductase, HP1164	190.98	4	0.00	0	0.00	0	
EEC24568.1	hypothetical protein, HP0563	190.92	2	210.98	2	0.00	0	[1,2]
QDY56806.1	outer membrane protein HofG, HP0914	185.87	5	432.31	9	587.01	9	[1,2]
EEC24488.1	outer membrane protein HopB, HP0913	171.29	3	226.23	3	192.94	3	[1,2]
WP_000960466.1	class II fructose-1,6-bisphosphate aldolase, HP0176	170.93	5	48.46	4	0.00	0	[1]
EEC25210.1	HP0018	165.11	2	0.00	0	0.00	0	[1,2]
EEC24174.1	hypothetical protein, HP0118	163.49	3	162.53	4	154.22	4	
EEC25339.1	hypothetical protein, HP0318	160.07	5	76.59	2	0.00	0	
EEC24687.1	translation elongation factor EF-G, HP1195	157.03	3	107.97	2	0.00	0	[1]
QDY56697.1	signal peptidase I LepB, HP0576	156.89	3	138.33	2	0.00	0	[1]
WP_000706040.1	fumarate reductase flavoprotein FrdA, HP0192	154.43	3	187.20	6	0.00	0	[1]
QDY57087.1	cag pathogenicity island protein, HP0545	154.09	5	246.42	7	176.06	6	[2]
WP_000885323.1	2-oxoglutarate ferredoxin oxidoreductase subunit beta, HP0590	153.15	4	106.59	3	49.71	2	
QDY56711.1	3-oxoacyl-ACP reductase FabG, HP0561	151.88	5	0.00	0	0.00	0	[1]
WP_001183642.1	fumarate reductase cytochrome b FrdC, HP0193	150.05	2	0.00	0	0.00	0	[1]
WP_000520987.1	molecular chaperone DnaK, HP0109	144.15	3	132.41	3	0.00	0	[1]
QDY56517.1	hypothetical protein, HP0781	141.73	2	258.63	5	102.83	3	[1,2]
WP_000743462.1	UPF0323 family lipoprotein, HP0232	141.24	4	372.88	8	391.53	6	[1,2]
EEC23875.1	hypothetical protein, HP1108	140.85	3	0.00	0	0.00	0	[1]
WP_001160549.1	fumarate hydratase FumC, HP1325	140.10	5	146.43	3	152.85	3	[1]
QDY55969.1	hypothetical protein, HP0367	137.51	4	0.00	0	151.44	4	[1,2]
WP_000813741.1	nucleoside-diphosphate kinase Ndk, HP0198	136.95	2	0.00	0	0.00	0	
QDY56411.1	molybdate ABC transporter substrate-binding protein, HP0473	135.89	3	137.68	2	165.31	3	[2]
EEC25054.1	predicted cytochrome c peroxidase, HP1461	135.01	4	333.48	8	76.29	4	[1,2]

QDY56580.1	acetolactate synthase, HP0709	133.74	3	75.68	3	0.00	0	
QDY56623.1	non-heme ferritin, HP0653	133.38	2	233.65	4	0.00	0	[1]
QDY55861.1	50S ribosomal protein L11, HP1202	130.27	4	0.00	0	0.00	0	[1]
WP_000467393.1	50S ribosomal protein L5, HP1307	128.40	3	80.19	2	0.00	0	[1]
QDY57056.1	outer membrane protein HofA, HP0209	122.04	4	217.56	5	284.40	4	[2]
QDY56627.1	aspartate ammonia-lyase, HP0649	119.99	5	0.00	0	0.00	0	[1]
QDY56769.1	DNA polymerase III subunit beta, HP0500	119.72	5	0.00	0	0.00	0	
QDY56735.1	cag pathogenicity island protein Cag1, HP0520	117.96	3	157.66	4	99.17	2	[1]
QDY56119.1	DUF1104 domain-containing protein; HP0129	113.30	3	406.21	5	320.59	5	[1,2]
QDY56376.1	aminopeptidase P family protein, HP1037	106.62	5	40.45	2	49.34	2	[1]
EEC24050.1	ribosome-associated trigger factor, HP0795	103.99	4	45.14	2	0.00	0	[1]
QDY56205.1	YbhB/YbcL family Raf kinase inhibitor-like protein, HP0218	102.04	3	52.65	3	126.05	3	
QDY57002.1	cytochrome bc complex cytochrome b, HP1539	101.65	2	123.08	2	0.00	0	[1]
QDY56386.1	transcriptional repressor Fur; HP1027	99.25	2	0.00	0	0.00	0	
EEC23825.1	hypothetical protein, HP0080	98.73	2	104.28	2	0.00	0	[1,2]
QDY55924.1	ATP synthase subunit gamma, HP1133	97.19	2	206.58	4	108.70	2	[1]
QDY56514.1	lipoprotein chaperone LolA, HP0785	95.16	2	215.81	4	132.12	2	[1,2]
EEC24507.1	beta barrel assembly factor BamD, HP1378	94.01	4	181.30	4	57.98	3	[1,2]
QDY57009.1	predicted lipoprotein, HP1546	93.58	2	111.07	2	153.73	3	[1,2]
QDY57021.1	class 1 fructose-bisphosphatase, HP1385	91.71	2	0.00	0	0.00	0	[1]
EEC25036.1	phospholipid synthesis protein PlsX, HP0201	91.21	7	77.59	3	0.00	0	[1]
EEC23979.1	glutamine synthetase GlnA, HP0512	89.79	3	0.00	0	0.00	0	[1]
EEC25112.1	phospholipid biosynthesis protein PlsY, HP1509	86.29	2	0.00	0	0.00	0	
QDY57014.1	preprotein translocase subunit YajC, HP1551	85.81	2	0.00	0	0.00	0	
QDY56151.1	Sel1-like repeat protein, HP0160	84.90	4	253.23	4	231.56	3	[1]
WP_001148292.1	YkgB family protein, HP0565	84.31	2	0.00	0	0.00	0	
QDY56183.1	enoyl-[acyl-carrier-protein] reductase FabI, HP0195	78.85	2	0.00	0	0.00	0	[1]
WP_001861250.1	outer membrane beta-barrel protein, HP1056	78.65	3	124.09	2	0.00	0	[1,2]
EEC24155.1	methyl-accepting chemotaxis protein, HP0103	77.49	3	291.98	6	0.00	0	[1]
EEC23926.1	outer membrane protein HofD, HP0487	77.49	2	0.00	0	0.00	0	[1,2]

QDY55747.1	50S ribosomal protein L4, HP1318	77.30	2	0.00	0	0.00	0	[1]
QDY55960.1	Sel1 repeat protein HcpC, HP1098	74.14	3	163.07	8	144.10	6	[2]
QDY55958.1	phosphogluconate dehydratase, HP1100	73.42	3	0.00	0	0.00	0	
QDY56407.1	oligoendopeptidase F, HP0470	72.52	2	0.00	0	0.00	0	[1]
QDY56761.1	cag pathogenicity island protein, HP0546	66.93	2	0.00	0	0.00	0	
WP_000037885.1	RNA polymerase subunit beta/beta', HP1198	65.11	2	0.00	0	0.00	0	
EEC25102.1	outer membrane protein HorK, HP1501	61.84	2	191.97	3	154.95	2	[1,2]
WP_000490795.1	cytochrome-c oxidase, cbb3-type subunit II, HP0145	61.09	3	186.57	5	77.56	3	
WP_000152120.1	NAD-binding protein, HP1398	60.06	5	41.83	2	0.00	0	[1]
QDY55836.1	cytochrome c-553, HP1227	59.60	2	234.05	5	139.62	5	[2]
QDY56465.1	flagellar motor accessory protein, HP0838	56.57	3	141.22	3	74.13	2	[2]
WP_000388019.1	2-oxoacid:acceptor oxidoreductase family protein, HP0591	54.58	2	0.00	0	0.00	0	[1]
WP_001160302.1	Hop family outer membrane protein	54.28	2	191.98	2	177.61	2	
EEC25132.1	hypothetical protein HP1527	54.18	3	0.00	0	0.00	0	[2]
QDY56646.1	Ni/Fe hydrogenase, HP0631	53.26	2	66.66	3	0.00	0	
WP_001169301.1	proline dehydrogenase family protein, HP0056	51.91	2	0.00	0	0.00	0	[1]
WP_001221685.1	IMP dehydrogenase, HP0829	44.24	4	0.00	0	0.00	0	[1]
QDY55772.1	RNA polymerase subunit alpha, HP1293	42.88	2					[1]
QDY55614.1	competence protein ComH, HP1527	0	0	598.85	13	209.04	5	[2]
QDY56572.1	DUF1104 domain-containing protein HP0719/HP0720	0	0	384.12	7	273.79	6	[2]
EEC25038.1	hypothetical protein HP0203	0	0	269.15	3	285.69	2	[2]
QDY56640.1	predicted lipoprotein HP0637	0	0	224.52	2	0.00	0	
WP_000770228.1	outer membrane beta-barrel protein, HP1055	0	0	172.83	2	0.00	0	[1]
WP_001861340.1	TrbG/VirB9 family protein, HP0040	0	0	145.70	3	141.27	3	
QDY56198.1	Sel1 repeat protein HcpA, HP0211	0	0	137.19	4	46.94	2	[2]
QDY55899.1	Flavodoxin FldA, HP1161	0	0	134.75	4	0.00	0	
EEC24849.1	TonB-system energizer ExbB, HP1339	0	0	134.01	3	0.00	0	
QDY56830.1	D-amino acid dehydrogenase DadA, HP0943	0	0	120.22	2	0.00	0	
QDY56175.1	hypothetical protein, HP0185	0	0	114.29	2	0.00	0	[1]

WP_000670506.1	DUF4006 family protein, HP0148	0	0	104.19	3	0.00	0	
QDY56307.1	sel1 repeat family protein	0	0	102.07	3	0.00	0	
EEC25328.1	hypothetical protein, HP0304	0	0	99.17	3	107.38	2	[2]
WP_000222336.1	flagellar sheath-associated autotransporter FaaA, HP0610/HP0609	0	0	0.00	0	337.80	6	[1]
QDY56333.1	outer membrane protein HofB, HP1083	0	0	96.31	2	334.86	6	[1,2]
QDY56385.1	DUF2147 domain-containing protein, HP1028	0	0	86.72	2	0.00	0	[2]
WP_000479939.1	outer membrane protein FrpB-1, HP0876	0	0	73.60	2	0.00	0	[1]
QDY56007.1	hypothetical protein, HP0408	0	0	72.31	2	0.00	0	[2]
QDY56714.1	hypothetical protein, HP0555	0	0	68.20	2	0.00	0	[1,2]
EEC24067.1	flagellar basal body protein FliL, HP0809	0	0	0.00	0	99.88	2	
EEC25307.1	vacuolating cytotoxin domain protein, HP0289	0	0	0.00	0	83.51	3	[1,2]
QDY56375.1	type II 3-dehydroquinate dehydratase AroQ, HP1038	0	0	55.12	2	69.16	2	[1]
QDY55920.1	ATP F0F1 synthase subunit B', HP1137	0	0	64.92	2	49.18	2	
QDY55691.1	TonB-system energizer ExbB homolog, HP1445	0	0	64.81	2	0.00	0	
EEC24916.1	hypothetical protein, HP0628	0	0	62.61	2	0.00	0	[1]
QDY56335.1	neuraminyllactose-binding hemagglutinin family protein, HP1081	0	0	61.36	2	0.00	0	
QDY56293.1	twin-arginine translocase subunit TatA, HP0320	0	0	54.65	2	0.00	0	
EEC25324.1	periplasmic solute-binding protein AppD, HP0301	0	0	48.26	2	0.00	0	[1]
EEC25048.1	hypothetical protein, HP1455	0	0	47.59	4	0.00	0	[2]
EEC25060.1	hypothetical protein, HP1467	0	0	47.11	2	0.00	0	[1]
QDY56171.1	CvpA family protein, HP0181	0	0	44.83	2	0.00	0	

[1]Indicate Mascot scores for proteins in the OMV samples prepared from wild-type *H. pylori* B128 and the Δ*hp0018* mutant strains H19 and H23.

[2]Indicate the number of peptide fragments generated following trypsin digestion for each protein.

[3]Identified as being significantly enriched in OMVs – 171 proteins (Zavan et al., 2019); 315 proteins (Olofsson et al., 2010)

Identified 226 proteins – 84% were identified by Zavan or Olofsson; 52% were identified by Zavan as proteins that were enriched in OMVs

1. Olofsson, A.; Vallstrom, A.; Petzold, K.; Tegtmeyer, N.; Schleucher, J.; Carlsson, S.; Haas, R.; Backert, S.; Wai, S.N.; Grobner, G.; et al. Biochemical and functional characterization of Helicobacter pylori vesicles. *Mol Microbiol* **2010**, *77*, 1539-1555, doi:10.1111/j.1365-2958.2010.07307.x.
2. Zavan, L.; Bitto, N.J.; Johnston, E.L.; Greening, D.W.; Kaparakis-Liaskos, M. Helicobacter pylori growth stage determines the size, protein composition, and preferential cargo packaging of outer membrane vesicles. *Proteomics* **2019**, *19*, e1800209, doi:10.1002/pmic.201800209.
